# Chromatin accessibility maps provide evidence of multilineage gene priming in hematopoietic stem cells

**DOI:** 10.1186/s13072-020-00377-1

**Published:** 2021-01-06

**Authors:** Eric W. Martin, Jana Krietsch, Roman E. Reggiardo, Rebekah Sousae, Daniel H. Kim, E. Camilla Forsberg

**Affiliations:** Institute for the Biology of Stem Cells, Department of Biomolecular Engineering, University of California, 1156 High Street, Santa Cruz, CA 95064 USA

## Abstract

Hematopoietic stem cells (HSCs) have the capacity to differentiate into vastly different types of mature blood cells. The epigenetic mechanisms regulating the multilineage ability, or multipotency, of HSCs are not well understood. To test the hypothesis that *cis*-regulatory elements that control fate decisions for all lineages are primed in HSCs, we used ATAC-seq to compare chromatin accessibility of HSCs with five unipotent cell types. We observed the highest similarity in accessibility profiles between megakaryocyte progenitors and HSCs, whereas B cells had the greatest number of regions with de novo gain in accessibility during differentiation. Despite these differences, we identified *cis*-regulatory elements from all lineages that displayed epigenetic priming in HSCs. These findings provide new insights into the regulation of stem cell multipotency, as well as a resource to identify functional drivers of lineage fate.

## Highlights

- HSCs have higher global chromatin accessibility than any unilineage progeny
- Megakaryocyte progenitors are the most closely related unipotent cell type to HSCs
- B cell commitment involves de novo chromatin accessibility
- Evidence of *cis-*element priming of lineage-specific genes in HSCs

## Introduction

**Fig. 1:**
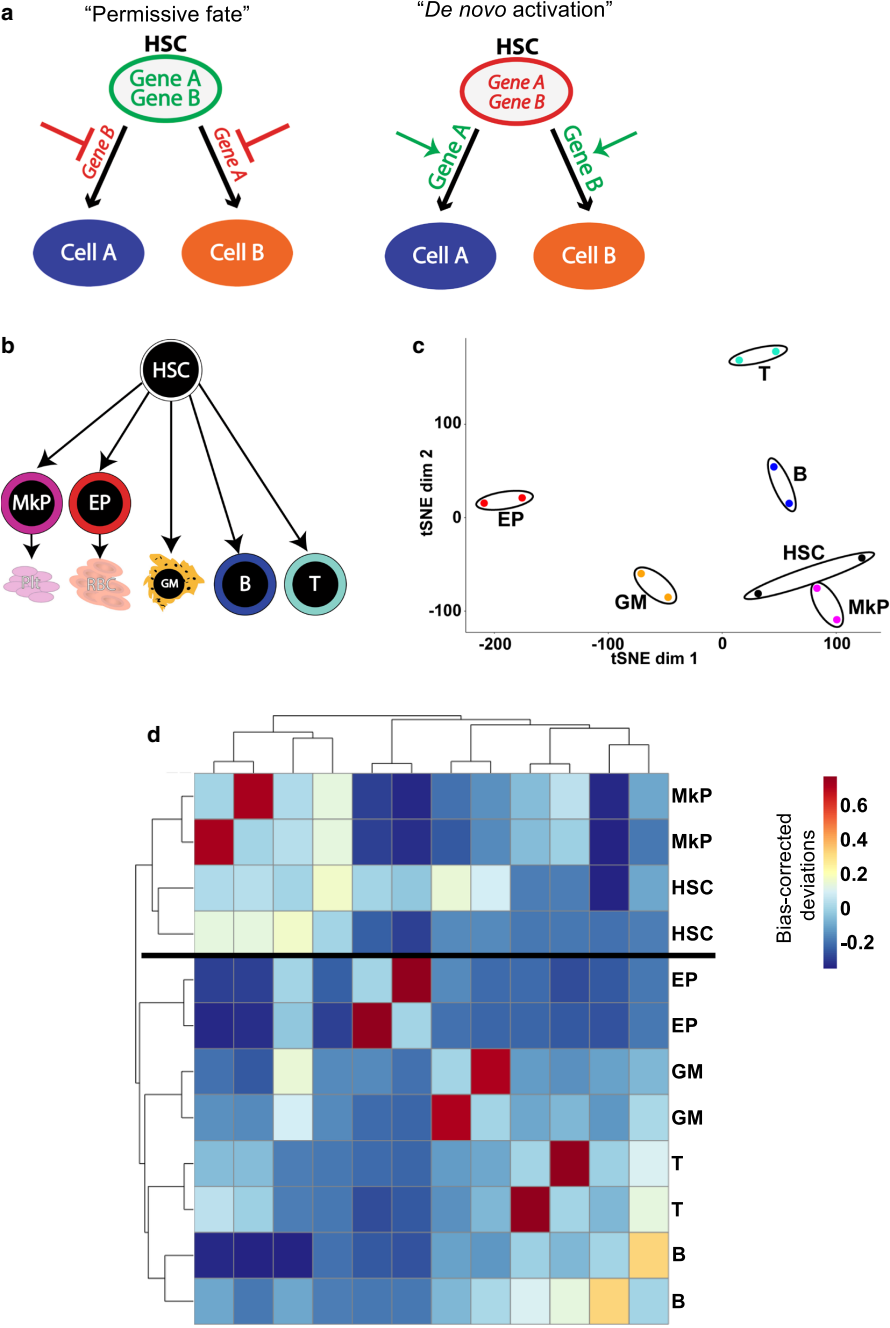
ATAC-seq maps of hematopoietic cell populations exhibit a high degree of reproducibility between replicates and a tight association of MkPs to HSCs. **a** Two models of epigenetic regulation of HSC fate. In the “permissive fate” model, CREs of lineage-specific genes of all possible lineage outcomes are in an accessible state (green) in HSCs, keeping genes “primed” for subsequent activation. After lineage commitment occurs towards one fate, the accessibility of primed elements of the alternative fate is restricted by epigenetic remodeling (red). In contrast, the “de novo activation” model posits that CREs of lineage-specific genes are in an inaccessible state (red) in HSCs, keeping genes silenced. Lineage commitment occurs by de novo decondensing of chromatin at the appropriate CRE, allowing for subsequent activation of the differentiation program (green). The CREs of alternative lineage fates remain epigenetically repressed (red). **b** Schematic diagram of the hematopoietic cells used in this study. Six cell populations were investigated: multipotent HSCs (Hematopoietic stem cells), unilineage MkPs (megakaryocyte progenitors) and EPs (erythroid progenitors), and mature GMs (Granulocyte/Macrophages), B cells, and T cells. **c** tSNE analysis of the ATAC-seq peaks revealed a high concordance of biological replicates. MkPs clustered close to HSCs, while EPs, GMs, B, and T cells separated across the tSNE plot. **d** Hierarchical clustering revealed high concordance of cell type-specific replicates. Similar to the tSNE analysis, MkPs clustered closest to HSCs. B and T cells were closely associated to each other but distant to HSCs, while GMs and EPs were contained within their own branches, closer to HSCs

Multipotency is a key feature of hematopoietic stem cells (HSCs) and essential for their ability to produce all types of blood and immune cells in situ and upon therapeutic stem cell transplantation. The mechanistic basis of multipotency is unclear, but previous studies have shown that the regulation of differentiation programs is achieved, in large part, through epigenetic remodeling of *cis*-regulatory elements (CREs) [17, 41, 46]. Thus, HSC multipotency may be enabled by accessible non-promoter CREs that keep loci competent for transcription factor binding and gene activation without active expression. Such selective “CRE priming” may underlie the developmental competence of specific cell types, which is then acted upon by inductive signals to gradually specify fate [45]. When all CREs that drive differentiation and lineage choice are primed in stem cells, that stem cell is in a permissive state **(**Fig. 1a**)** and is competent to initiate differentiation into all mature lineages.

We sought to test two models of HSC multipotency that are based on regulation of chromatin organization: the “permissive fate model” and a “de novo activation model” (Fig. 1a). Supporting a role for the permissive model in stem cell lineage potential are observations of bivalent histone domains that maintain key developmental genes in embryonic stem cells (ESCs) poised for activation [3], and an overall accessible chromatin state in both ESCs and HSCs compared to lineage-restricted progenitors and mature cells [19, 20, 44]. When differentiation occurs, the genes poised for differentiation into the induced lineage are activated while CREs that would drive differentiation into alternative lineages are silenced. This has been observed in ESCs and during differentiation of ESCs into endoderm [46, 47]. Our observation of global chromatin condensation and localization of H3K9me3-marked repressed domains or heterochromatin towards the nuclear periphery during HSC differentiation also support the permissive model [44]. Inversely, in the de novo activation model (Fig. 1a), CREs that drive lineage fate are inaccessible in HSCs. Differentiation and lineage choice occur by “unlocking” these CREs. Transcriptional and functional analyses of hematopoietic stem and progenitor cells support this de novo model, where lymphoid potential is *gained* in progenitor cells rather than being a consequence of CRE priming in HSCs [6, 12, 18, 32].

In order to interrogate these models and how they pertain to the regulation of competence in hematopoiesis, as well as gain a better understanding of the relationships between epigenetic, transcriptomic and functional observations, we mapped global chromatin accessibility using the Assay for Transposase Accessible Chromatin by High Throughput Sequencing (ATAC-seq) [8]. This assay allows assessment of high resolution, genome-wide chromatin accessibility throughout differentiation programs of rare cells. The dynamics of chromatin accessibility in erythro-megakaryopoiesis [24] and granulocyte/macrophage development [9] have been highly informative. From these studies, the bulk observations gave us insight into the dynamics of lineage commitment during hematopoiesis, while single-cell analysis revealed the heterogeneity of epigenomic states and, therefore, lineage bias in progenitors throughout hematopoiesis. Based on those studies, as well as reports of global chromatin accessibility of embryonic [3, 10, 20] and hematopoietic [11, 44] stem cells, we hypothesized that HSCs are in a permissive chromatin state where CREs that control fate decisions are primed in HSCs. Here, we tested this hypothesis by performing in-depth ATAC-seq investigation of HSCs and 5 unipotent lineage cell populations representing the five main hematopoietic lineages (Fig. 1b), as defined by previously published phenotypes [4, 6].

## Results

### Mapping of chromatin accessibility in HSCs and unipotent lineage cells identified a tight association of megakaryocyte progenitors to HSCs

To determine the dynamics of genome accessibility throughout hematopoiesis, we sorted six primary hematopoietic cell types (Fig. 1b) and performed ATAC-seq of libraries with expected fragment size distributions (Additional file 1: Figure S1) [8]. We identified 70,731 peaks in HSCs, 47,363 peaks in megakaryocyte progenitors (MkPs), 38,007 in erythroid progenitors (EPs), 30,529 in granulocyte/macrophages (GMs), 70,358 in B cells, and 51,832 in T cells (Table 1). From these peak-lists we combined and filtered the peaks using the chromVAR package to only the most significant peaks, as defined by [38] and identified a total of 84,243 peaks, referred to as the master peak-list throughout the study (Table 1). To assess data quality, we analyzed replicate clustering and cell type relationships of all 6 cell types using principal component analysis and dimensionality reduction as a t-Distributed Stochastic Neighbor Embedding (tSNE) plot [38]. All biological replicate samples closely associated with each other by tSNE analysis (Fig. 1c), as well as by hierarchical clustering using the chromVAR output (Fig. 1d). We observed two primary clusters in Fig. 1d: an HSC/MkP cluster and all other cell types. We also observed a distinct lymphoid cell subcluster containing only B and T cells, while GMs and EPs clustered independently. MkPs have the most similar accessibility to HSCs, with the ranking of the other cell types from most to least similar as EPs, GMs, Bs and then Ts. This is consistent with our tSNE analysis (Fig. 1c), where HSCs and MkPs closely associated with each other, and with studies that have reported a close relationship of HSCs with the megakaryocyte lineage [14, 37] and that erythropoiesis requires chromatin remodeling for differentiation to occur [24].

**Table 1:** Peak counts and peak distribution relative to protein-coding gene promoters in each cell type

Cell type	ATAC peaks	Promoter peaks (± 500 bp of TSS	Non-promoter peaks		
			Coding (exons + TTS + TSS)	Introns	Intergenic
Master peak-list	84,243	13,171	5243	34,137	31,692
HSC	70,731	27,973	4166	18,931	19,661
MkP	47,363	23,998	2013	10,036	11,316
EP	38,007	23,243	2014	7040	5710
GM	30,529	15,559	1440	6697	6833
B	70,358	24,596	4461	21,210	20,091
T	51,832	25,103	2016	11,929	12,784

### Visualization and comparison of ATAC-seq data generated in this study correlated with known expression patterns of cell type-specific genes

**Fig. 2:**
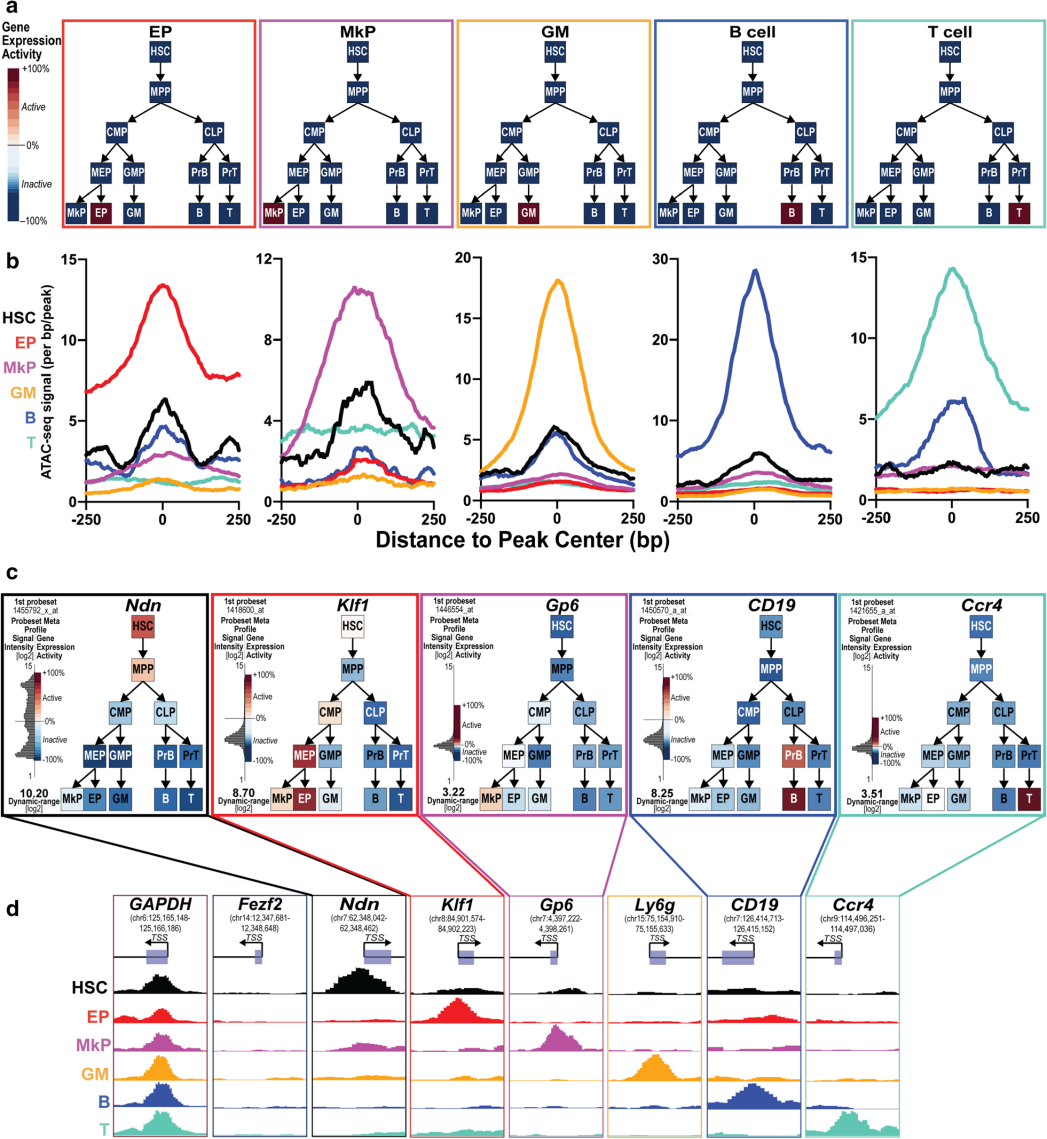
Promoter accessibility correlated with known expression patterns of cell type-specific genes. **a** Lineage-specific gene expression patterns used to find all genes expressed within each unipotent lineage cell type. The level of expression (red = high; blue = low/not expressed) according to the Gene Expression Commons (GEXC) database. **b** Lineage-specific promoters had accessibility of the corresponding unipotent lineage cell types. Homer histograms of the average cumulative signal of all cell types used in this study across the lineage-specific promoter gene lists for EPs, MkPs, GMs, B cells, and T cells. **c** Lineage-specific expression of one example gene each for MkPs, EPs, B, or T cells. The level of expression (red = high; blue = low/not expressed) according to the Gene Expression Commons (GEXC) database of an example gene with cell type-specific ATAC-seq promoter peak. The probeset for the GM-specific *Ly6g* is not present in GEXC and therefore not displayed. **d** Cell type-specific chromatin accessibility visualized as ATAC-seq read-counts at transcription start sites (TSS) using UCSC Genome Browser snapshots. Depiction of the six ATAC-seq libraries used in this study with example genes that had ATAC-seq signal in all samples (*GAPDH*; positive control), no samples (*Fezf2*; negative control), or in a specific cell type: HSCs (*Ndn*), EPs (*Klf1*), MkPs (*Gp6*), GMs (*Ly6g*), B cells (*CD19*), and T cells (*Ccr4*)

As another assessment of the quality and reproducibility of our ATAC-seq data, we used the Gene Expression Commons (GEXC) expression database [40] to generate a list of genes that were expressed only in each unipotent lineage cell type (Fig. 2a). From each list, we calculated the normalized average signal centered at the promoter of each cell-type-specific peak-list for each cell type by generating histograms using HOMER [22] (Fig. 2b). We observed the expected cell type-specific accessibility for each unipotent lineage with minimal signal from the other cell types. In addition, we visualized the ATAC-seq signals across promoters of some example genes with known cell type-specific expression patterns, plus a negative (expressed in none of the cell types) and a positive (expressed in all of the cell types) control: *Gapdh* (expressed in all cell types)*, Fezf2* (not expressed in any cell type)*, Ndn* (expressed in HSCs only)*, Klf1* (EPs only)*, Gp6* (MkPs only)*, Ly6g* (GMs only), *CD19* (B cells only), and *Ccr4 *(T cells only) (Fig. 2c, d). *Ly6g* was not available in GEXC but is a well-known GM-selective gene [23]. We observed the expected accessibility peaks in each cell type, as well as a minimal signal from cell types without expression of those genes (Fig. 2d). As an example of a well-characterized locus, we visualized our ATAC-seq data across the mouse β-globin cluster. As expected, we observed EP-selective accessibility of the hypersensitive sites in the locus control region (LCR) and of adult globin gene promoters β*-major* and β*-minor* [30, 34] (Additional file 2: Figure S2). The overall high level of reproducibility between independent sample replicates and clustering strategies (Fig. 1c, d), as well as the expected accessibility in cell type-specific genes (Fig. 2, Additional file 2: Figure S2), indicated that we had generated high-quality chromatin accessibility maps of these 6 cell types.

### HSCs have greater global accessibility and undergo more extensive chromatin remodeling upon lymphoid differentiation

**Fig. 3:**
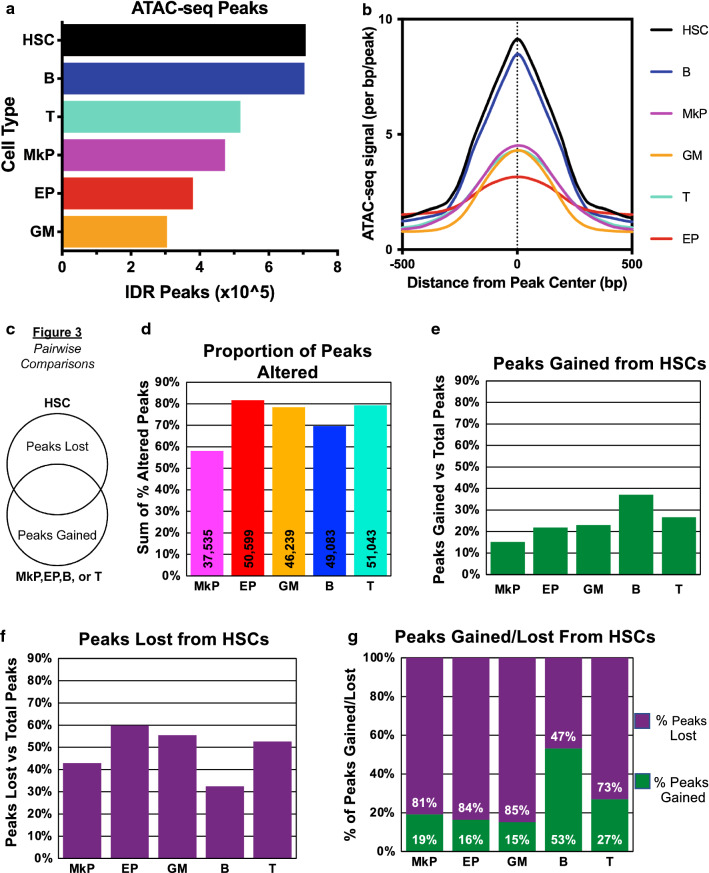
Greater overall global accessibility of HSCs and more extensive chromatin remodeling upon lymphoid differentiation. **a** HSCs had the highest number of peaks of all hematopoietic cell types. The total number of individual peaks are displayed for each cell type. HSCs had the highest number of peaks followed by B cells, T cells, MkPs, EPs, then GM cells. **b** HSCs had the highest total accessibility signal across all peaks of all hematopoietic cell types analyzed. Average cumulative signal across the master peak-list (the number of sequencing reads that fall into the detected peaks) was determined by the -hist function of HOMER annotatePeaks.pl. **c–g** Comparisons of the number of peaks gained and lost upon HSC differentiation into unipotent cells revealed that MkPs had the most similar accessibility profile to HSCs. **c** Schematic of the pairwise comparisons made. HSC peaks were compared with one unilineage cell type at a time and those comparisons are reported in **d**–**g**. **d** MkPs had the lowest percentage of altered peaks from HSCs compared to the other 4 unilineage cell types. The percentage of all non-overlapping peaks (peaks both gained and lost) calculated as the ratio of unique peaks in each cell type when compared pairwise to HSCs divided by the total number of peaks called in that cell type are displayed here. The numbers in the bars represent the total number of peaks altered (gained + lost) for each cell type. EPs had the highest percentage of peaks altered (gained + lost), followed by T cells, GMs, then B cells. **e** B cells had the highest percentage of peaks gained from HSCs, while MkPs had the lowest. Calculations as in **d**, but only peaks gained are shown. **f** EPs had the highest percentage of peaks lost from HSCs, while B cells had the least. Calculations as in **d**, but only peaks lost are shown. **g** B cells were the only lineage with more peaks gained (53%) than lost (47%) upon differentiation from HSCs. In this panel, the sum of peaks gained and lost in each cell type was set to 100% and then the ratio of peaks gained and lost was displayed. T cells had the second highest proportion of peaks gained (27%), followed by MkPs (19%), EPs (16%), then GMs (15%)

Using a number of quantitative, but non-sequence-specific assays, we previously reported that chromatin is progressively condensed upon HSC differentiation into unilineage and mature cells [44]. To test whether the ATAC-seq data recapitulated these findings, we quantified the total number of distinct peaks, as well as the cumulative read-counts for all peaks, for each cell type. First, we took each cell type’s optimal peak-list from the Irreproducible Discovery Rate (IDR) analysis [31] and reported the number of peaks. We observed the highest number of peaks in HSCs (Fig. 3a), closely followed by B cells. In parallel, we quantified global accessibility by calculating the normalized average signal over the master peak-list for each cell type by generating histograms using HOMER [22]. We observed similar ordering compared to the peak number, with HSCs having the highest average signal and B cells the second highest (Fig. 3b). The low signal in EPs is possibly due to widespread transcriptional silencing as the next step towards becoming highly specialized red blood cells and ejection of nuclei [1]. Although these measurements are not completely independent, there is not a strict correlation between peak count and cumulative peak signal: for example, compared to EPs, GMs have fewer peaks (Fig. 3a) but higher cumulative readcount (Fig. 3b). Interestingly, HSCs displayed both the highest number of peaks and the greatest peak signal. These results are consistent with our previous findings of progressive chromatin condensation upon HSCs differentiation [44].

### Comparisons of peaks gained and lost as HSCs differentiate into unilineage cells revealed an overall gain of accessibility selectively for B cell differentiation

To assess the number of peaks that changed upon HSCs differentiation, we took the IDR optimal peak-list for each cell type and performed pairwise comparisons between HSCs and the five mature/unipotent cell types (Fig. 3c). We quantified the number of peaks gained and lost by the unipotent progenitors/mature cells compared to HSCs (Fig. 3c–g). MkPs had the lowest number of peak changes (peaks gained plus lost; Fig. 3d), and therefore have the greatest proportion of peaks in common with HSCs. This was primarily driven by the low percentage of peaks gained (Fig. 3e), as opposed to peaks lost (Fig. 3f) upon HSC differentiation into MkPs. In contrast, EPs had the highest percentage of total peaks changed (Fig. 3d) due to the greatest percentage of peaks lost (Fig. 3f). This could be driven by EPs starting to shut down transcription to become highly specialized and eject their nuclei, reflected by the overall low accessibility observed (Fig. 3a, b). B cells had the highest percentage of peaks gained and the lowest percentage of peaks lost compared to the other cell types (Fig. 3e, f) and was the only cell type where the percentage of peaks gained was higher than peaks lost (Fig. 3g). This suggests that B cell fate requires chromatin remodeling to open up sites that drive B cell lineage fate.

### Exclusively shared peaks between HSCs and unipotent cell types are primarily non-promoter and are enriched for known cell-type-specific transcription factors

**Fig. 4:**
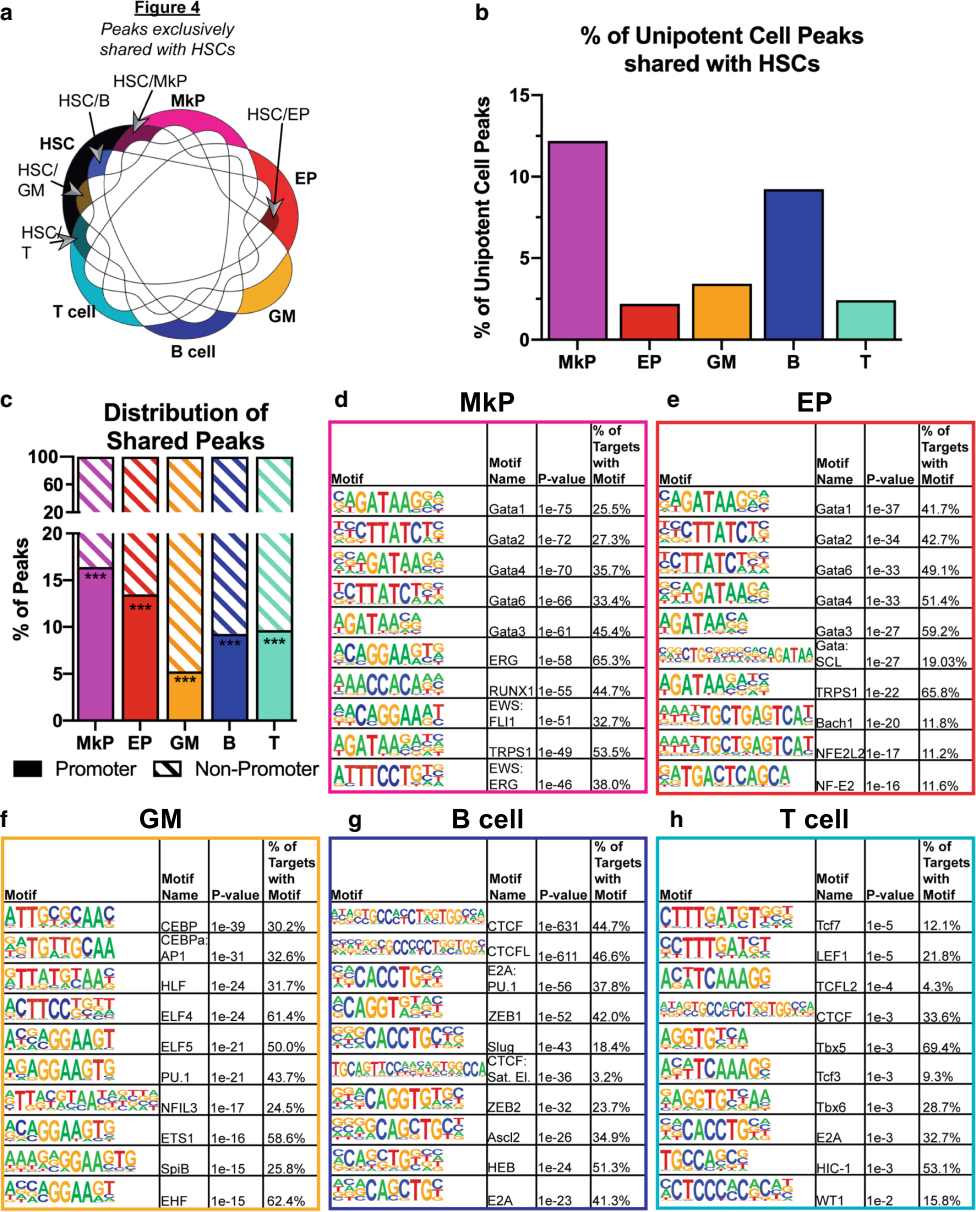
Peaks shared between HSCs and unipotent cell types are primarily non-promoter and are enriched for known cell type-specific transcription factors. **a** Schematic for how the unipotent lineage peaks exclusively intersected with HSC peaks were generated. Peaks were compared using HOMER mergePeaks.pl tool using peak-lists from the 6 cell types assayed. The resulting 5 overlapping peak-lists contained shared peaks between HSCs and only the unipotent cell type of interest (but not present in any of the other four lineages). The five exclusive pairwise comparisons (e.g., HSC/MkP only, HSC/EP, etc.) were used for panels **b–h**. **b** MkPs have the highest peak overlap with HSCs. The number of unipotent lineage peaks that were uniquely intersected with HSCs was divided by the total number of peaks for each mature cell type. MkPs had the highest percentage of HSC overlap (12.2%), followed by B cells (9.2%), GMs (3.4%), T cells (2.4%), then EPs (2.2%). **c** Peaks exclusively shared between each unipotent cell type and HSCs were significantly enriched in the non-promoter regions of the genome. The shared peak-lists described in **a** were annotated using HOMER annotatePeaks.pl function and filtered as promoter (± 500 bp from TSS), and non-promoter (< -500 bp and >  + 500 bp from TSS). The number of promoter and non-promoter peaks was divided by the total number of peaks for each cell type. For all cell types, less than 20% of peaks were promoter peaks, with MkPs with the highest (16.4%) and GMs with the lowest (5.3%) percentage. This is a significant (< 0.001) difference compared to the normal distribution of promoter peaks (35–61%) for each cell type assayed. ***p-value of < .001. **d–h** Unipotent lineage peaks exclusively intersected with HSC peaks displayed enrichment of motifs for transcription factors with known roles in lineage differentiation. Motifs were found using HOMER findMotifsGenome.pl function, with a background file containing the combined peak-lists of the other 4 cell types. The top 10 results, as ranked by p-value from the known_motifs.html output, are shown. **d** In MkP/HSC peaks, Gata family peaks made up 5 of the top 10 hits, followed by ERG, Runx1, and fusions EWS:FL1 and EWS:ERG. **e** EP/HSC-enriched motifs also contained Gata factors, as well as the combination Gata:SCL motif and the known beta-globin locus control binder NFE2 and its paralog NFE2L2. **f** GM/HSCs had CEBPa and PU.1 motifs as top hits, along with ETS transcription factor binding sites. **g** B cell/HSC-enriched motifs had CTCF with CTCFL (BORIS) as the top two hits. B cells/HSC peaks also had E2A motifs enriched, as well as Ascl2, Slug, and ZEB1/2. **h** Tcf7 motif was the top hit for T cell/HSC-shared peaks, along with CTCF and Tbx5/6. Similar to the B-cell/HSC list, the T-cell/HSC list was also enriched for E2A motifs

We then turned our attention from peaks that were different between HSCs and their progeny to instead focus on elements with shared accessibility. We hypothesized that peaks that are exclusively shared between HSCs and one unipotent cell type contain elements that drive lineage commitment into that cell type. We filtered the peak-lists of all 6 cell types against each other using the HOMER mergePeaks.pl tool and annotated the peak-lists that each of unipotent lineage cell types exclusively shared with HSCs (Fig. 4a). We quantified the percentage of peaks that each unipotent cell type shared with HSCs (Fig. 4b). Consistent with the clustering profiles (Fig. 1c, d), MkPs had the highest percentage of peaks that were shared exclusively with HSCs. This similarity appeared to be primarily manifested in non-promoter elements: we annotated the exclusively shared peaks and categorized them as promoter or non-promoter peaks (Fig. 4c) and compared the distributions to the annotated peak-lists for each cell type assayed (Table 1). All of the exclusively shared peak-lists had significant enrichment (*p*-value < 0.001) of non-promoter peaks compared to the normal distribution of peaks in our dataset. Thus, non-promoter elements were shared between HSCs and their progeny significantly more frequently than promoter elements, especially with MkPs. Many, but likely not all, of these non-promoter accessible sites may serve as enhancers: about one-third of the non-promoter peaks overlapped with an enhancer catalog generated from chromatin immunoprecipitation (ChIP) experiments in blood cells [27] (Additional file 3: Figure S3A). Similar levels of overlap was observed between the ATAC-accessible peaks in our ATAC exclusively shared peak-lists with H3K4me1 modifications in HSCs, while less overlap was observed for H3K27Ac, at the aggregate and cell type-specific level (Additional file 3: Figure S3B, C).

To determine what transcription factor binding sites were present within the exclusively shared peaks, we performed motif enrichment using the HOMER package and reported the top 10 results for each cell type, sorted by *p*-value (Fig. 4d–h). The peaks that HSCs shared with MkPs (Fig. 4d) or EPs (Fig. 4e) were primarily enriched for Gata family transcription factors and their inhibitor TRPS1. Notably, HSC/MkP peaks also had enrichment of ERG and Runx1, which are known drivers of hematopoiesis [21, 26]. For HSC/EPs, Gata1 was the most enriched motif, with the Gata:SCL combination motif and NF-E2 and NFE2L motifs also scoring in the top ten. These factors are all known to be important in red blood cell differentiation, and NF-E2 is known to regulate SCL and Gata2 [42]. HSC/GM peaks had enrichment of known regulators of GM cell fate, such as CEBP, PU.1, and SpiB (Fig. 4f). HSC/B cells primarily had CTCF and CTCFL motif enrichment (Fig. 4g). These motifs could be a reason for the overall high number of peaks observed in B cells (Fig. 3a, b), as 44.7% and 46.6% of the shared peaks contained CTCF or CTCFL motifs, respectively. HSC/T cell peaks were enriched for Tcf and Tbx family factors that are known to play a role in T cell development (Fig. 4h). Overall, all five HSC-shared peak-lists had enrichment of transcription factors that are known to be important for normal differentiation for each lineage.

### Evidence of *cis-*element priming of lineage-specific genes in HSCs

**Fig. 5:**
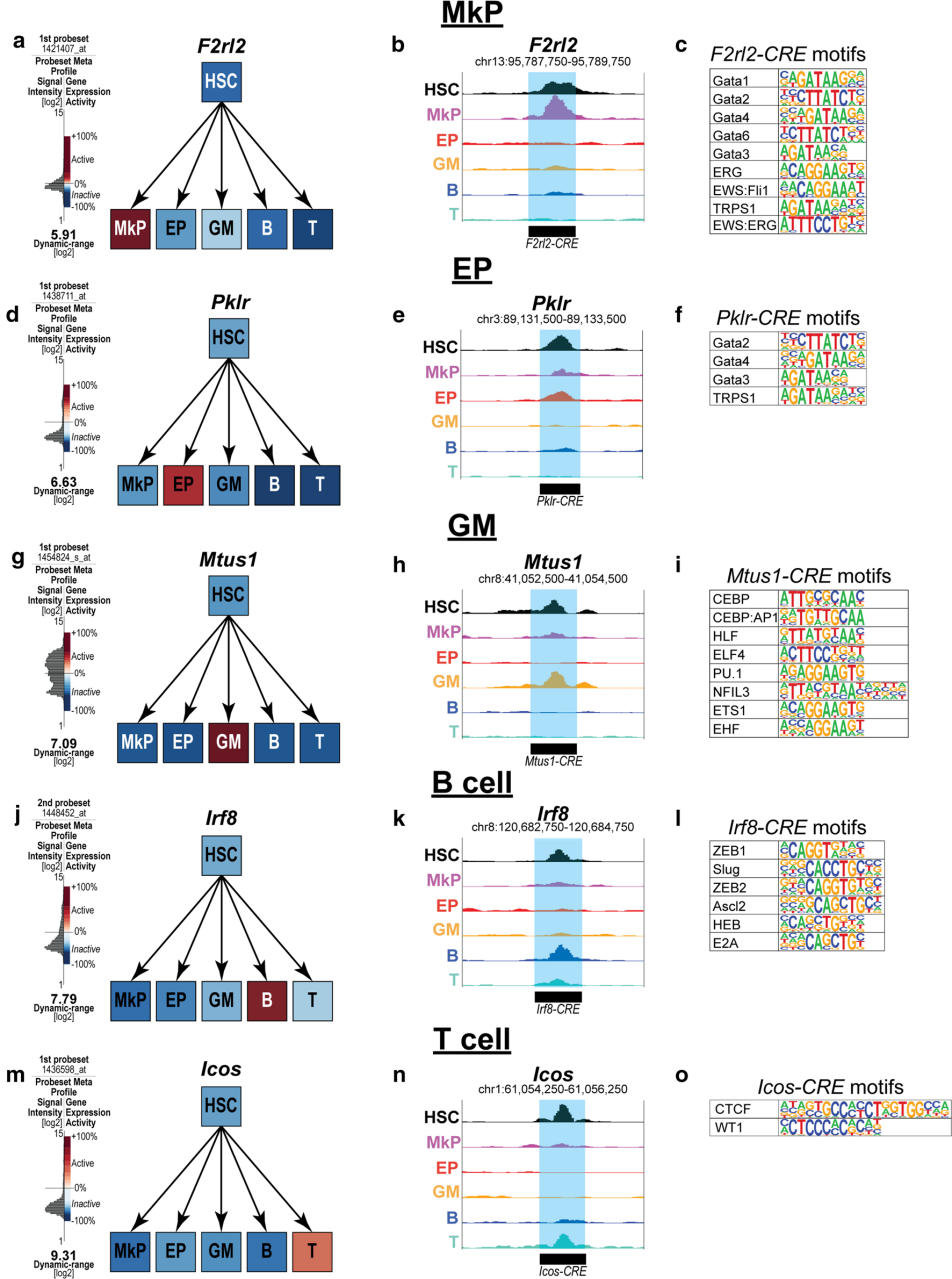
Examples of *cis-*element priming of lineage-specific genes in HSCs. **a** GEXC expression data reported expression of *Thrombin receptor like 2 (F2lr2)* selectively in MkPs. **b** A *cis-*element predicted to be associated with *F2rl2* by GREAT was accessible in both MkPs and HSCs, but not in any other unipotent cell type. **c** The *F2rl2* CRE contained the transcription factor binding motifs for 9 out of the top 10 enriched motifs in MkPs. The only motif not present is Runx1. **d** GEXC expression data reported expression of *Pyruvate kinase liver and red blood cell (Pklr*) in EPs, and not any other cell type. **e** A *cis-*element predicted to be associated with *Pklr* by GREAT was accessible in both EPs and HSCs, but not in any other unipotent cell type. **f** The *Pklr* CRE contained the binding motifs for Gata2, Gata4, Gata3 and TRPS1. **g** GEXC expression data reported selective expression of *Mitochondrial tumor suppressor 1* (*Mtus1*) in GMs and no expression in any other cell type. **h** A *cis-*element predicted to be associated with *Mtus1* by GREAT was accessible in both GMs and HSCs. **i** CEBP, CEBP:AP1, HLF, PU.1, NFL3, ETS1, and EHF binding motifs were present in the *Mtus1* CRE reported in **h**. **j** GEXC expression data reported *Interferon regulatory factor 8 (Irf8)* expression only in B cells, not in the other unipotent lineage cells or in HSCs. **k** A *cis-*element predicted by GREAT to be associated with *Irf8* was accessible in both B cells and HSCs. **l** ZEB1/2, Slug, Ascl2, HEB, and E2A binding motifs were found within the *Irf8* CRE displayed in **k**. **m** GEXC expression data reported *Inducible T cell co-stimulator (Icos)* expression only in T cells, but not in the other unipotent lineage cells or HSCs. **n** A *cis-*element predicted by GREAT to be associated with *Icos* was accessible in both T cells and HSCs. **o** CTCF and WT1 motifs were found within the *Icos* CRE displayed in **n**

Previous work on understanding multipotency and developmental competence suggests a model where competence is conferred by transcriptional priming: being competent of transcription factor binding and gene expression, without active expression [25]. One of the suggested regulators of transcriptional priming are non-promoter *cis-*regulatory elements (CREs). This means that CREs that drive lineage fate for all lineages are accessible in HSCs in our permissive fate model and inaccessible in our de novo activation model. We hypothesized that CREs that are exclusively shared between HSCs and a unipotent lineage cell are potential drivers of that lineage. We utilized the GREAT tool [33] to annotate and predict the target genes for each exclusively shared CRE. Here we report examples of genes and a predicted CRE for each lineage that is primed in HSCs. In addition, we linked the motif enrichment with the GREAT analysis by annotating the CREs using the top 10 motifs enriched by p-value (Fig. 4d–h) for each exclusive HSC/unipotent cell type. In MkPs, a predicted CRE for *Thrombin receptor like 2* (*F2rl2*) was found. This gene is expressed only in MkPs (Fig. 5a), while the CRE is only accessible in HSCs and MkPs (Fig. 5b). This CRE contained 9 out of the top 10 motifs, with the Runx1 motif being the only one missing (Fig. 5c). *Pyruvate kinase liver and red blood cell* (*Pklr)* was found to be expressed only in EPs (Fig. 5d), and a predicted CRE was accessible only in HSCs and EPs (Fig. 5e). Motifs for Gata2, Gata3, Gata4, and TRPS1 were found within the CRE (Fig. 5f). In GMs, *Mitochondrial tumor suppressor 1* (*Mtus1*) was found to be primed in HSCs, with expression only in GMs (Fig. 5g), accessibility of a predicted CRE only in HSCs and GMs (Fig. 5h), and the presence of transcription factors known to play a role in GM development, such as CEBP and PU.1 (Fig. 5i). In B cells, *Interferon regulatory factor 8* (*Irf8*), is only expressed in B cells (Fig. 5J), the predicted CRE is only accessible in both B cells and HSCs (Fig. 5k), and contained 5 out of the top 10 motifs, ZEB1/2, Slug, Ascl2, HEB, and E2A (Fig. 5l). In T cells, the gene *Inducible T cell co-stimulator (Icos)* is only expressed in T cells (Fig. 5m), a predicted linked CRE is accessible in both T cells and HSCs (Fig. 5n) and contains motifs for CTCF and WT1 (Fig. 5o). Taken together, these examples represent CRE priming in HSCs, along with the corresponding transcription factors that may act on each element to guide HSC fate.

## Discussion

### MkPs and HSCs have the most similar accessibility profile

Here, we compared the genome-wide accessibility by ATAC-seq of the multipotent HSCs and unipotent lineage cell types (EPs, MkPs, GMs, B, and T cells). Through hierarchical clustering analysis, we observed erythromyeloid and lymphoid relationships that are consistent with the classical model of hematopoiesis (Fig. 1d) [4, 7, 28, 39]. By both PCA and hierarchical clustering, we observed that MkPs were the most similar to HSCs based on their accessibility profiles (Fig. 1). This relationship is reflected in a high level of overlap of peaks, as MkPs had the fewest peaks gained or lost from HSCs compared to the other cell types (Fig. 3) and had the largest percentage of peaks exclusively shared with HSCs (Fig. 4b). These findings are in agreement with recent clonal studies of hematopoiesis that reported a megakaryocyte lineage bias of HSCs [14, 37]. According to hierarchal clustering, EPs had the second closest association to HSCs (Fig. 1d) possibly supporting erythropoiesis as the default fate for hematopoiesis [6] under conditions where chromatin remodeling silences megakaryocyte driver elements [24]. On the other end of the spectrum, the least similar cell types to HSCs were the lymphoid cell types (Fig. 1d). This greater difference was primarily due to a high proportion of peaks gained (Fig. 3e) rather than lost (Fig. 3f) upon differentiation from HSCs, leading to a greater ratio of peaks gained:lost for lymphoid cells than for erythromyeloid lineages (Fig. 3g).

### Evidence of multilineage priming in HSCs

The priming of genes for transcription likely initiates within CREs, which can then drive the activation of promoter targets. These enhancers can act as drivers of lineage fate [46] and their accessibility is a putative regulator of competence in stem cells. We made the assumption that peaks that are exclusively shared between HSCs and the unipotent lineage cells contain CREs that are specific for driving differentiation into that lineage. We observed that the majority of exclusively shared peaks were non-promoter peaks (Fig. 4b) and were enriched for binding motifs of transcription factors known to be important for differentiation into each lineage (Fig. 4d–h). The enrichment of binding sites for known lineage-specific transcription factors suggests that many of the accessible sites may play functional roles. Additionally, about one-third of the exclusively shared ATAC peaks were enriched for the H3K4me1 histone modification, which is linked to a primed enhancer state [13], indicated that a subset are likely functional enhancers (Additional file 3: Figure S3); other ATAC-accessible elements may mark transcription start sites for non-coding genes, which are abundant and highly tissue-specific in the mouse genome [36]. By using the GREAT tool, we made predictions for the target genes for the many ATAC-identified putative CREs that were present in the HSC/mature cell exclusive lists. The examples shown in Fig. 5 provide evidence that multilineage priming exists in HSCs.

### Both permissive and de novo epigenetic mechanisms influence hematopoiesis

Analogous to other stem cell systems, multipotent HSCs with the competence to differentiate into diverse cell types reside at the top of the blood cell hierarchy. We tested two potential models of the mechanism of multipotency, the permissive fate and de novo activation (Fig. 1a). We found evidence for both. Supporting the permissive fate model are the observations that HSCs had the highest global accessibility (Fig. 3a/b), that peaks were lost in every unipotent cell type from HSCs (Fig. 3f), that every unipotent cell type shared some peaks exclusively with HSCs (Fig. 4b), and that evidence of multilineage priming of CREs were found in HSCs (Fig. 5). The de novo activation model was supported by the observation that new peaks were gained during differentiation into all five lineages (Fig. 3e), and previous studies reporting progressive upregulation of lineage-specific genes as HSCs transition into progenitors [18, 43]. Interestingly, in the β-globin locus, HS2, the strongest enhancer of globin expression [2, 16]], was highly accessible in HSCs, whereas the other HSs were not (Additional file 2: Figure S2). Thus, “priming” of this locus may occur in HSCs via HS2 (adhering to the permissive model of Fig. 1a), followed by induced accessibility (de novo model, Fig. 1a) of the other HSs and active β-globin expression upon erythroid differentiation. Thus, both permissive and de novo mechanisms likely influence hematopoietic fate decisions. Interestingly, we found evidence that the balance between the two models varies between lineages. For example, B cells, and to a lesser extent T cells, had a higher proportion of peaks gained than lost compared to erythromyeloid lineages (Fig. 3g). This may indicate that the megakaryocyte/erythroid lineage is in a more primed state in HSCs, whereas lymphopoiesis requires more extensive chromatin remodeling to both prime lymphoid CREs not accessible in HSCs and simultaneously shut down the megakaryocyte/erythrocyte trajectory. The cell output and kinetics from in vivo lineage tracing and reconstitution assays support these conclusions [4–6, 14, 37, 48]. Our identification of specific, putative regulatory CREs will enable functional testing of these elements.

## Experimental procedures

### Mice and cells

All experiments were performed using 8- to 12-week-old C57BL/6 wild-type mice in accordance with UCSC IACUC guidelines. Hematopoietic cells were isolated from BM by crushing murine femurs, tibias, hips, and sternums as previously described [35]. Stem and progenitor cell fractions were enriched using CD117-coupled magnetic beads (Miltenyi). Cells were stained with unconjugated lineage rat antibodies (CD3, CD4, CD5, CD8, B220, Gr1, Mac1, and Ter119) followed by goat-α-rat PE-Cy5 (Invitrogen). Stem and progenitor cells were isolated using fluorescently labeled or biotinylated antibodies for the following antigens: cKit (2B8, Biolegend), Sca1 (D7, Biolegend), Slamf1(CD150) (TC15-12F12.2, Biolegend), CD41(MWReg30, Biolegend), and CD71(RI7217, Biolegend). Cells were sorted using a FACS Aria II (BD Bioscience). HSCs were defined as cKit^+^ Lin^−^ Sca1^+^ Flk2^−^ and Slamf1^+^; MkPs as cKit^+^Lin^−^Sca1^−^Slamf1^−^CD41^+^. Unipotent lineage cells were isolated by the following markers and as described previously [15, 29]: EPs, Lin(CD3, CD4, CD5, CD8, B220, Gr1, and Mac1)^−^ CD71^+^Ter119^±^; GMs, Lin(CD3, CD4, CD5, CD8, B220, and Ter119)^−^ Gr1^+^Mac1^+^ (“GM” cells were positive for both Gr1 and Mac1); T cells, Lin(CD5, B220, Gr1, Mac1, and Ter119)^−^ CD25^−^CD3^+^CD4^±^CD8^±^; B cells, Lin(CD3, CD4, CD8, Gr1, Mac1, and Ter119)^−^CD43^−^B220^+^.

### ATAC-seq

ATAC-seq was performed as previously described [8]. Briefly, cells were collected after sorting into microcentrifuge tubes containing staining media (1xDPBS,1 mM EDTA with 5% serum). They were centrifuged at 500×*g* for 5 min at 4 ˚C to pellet the cells. The supernatant was aspirated, and the cells were washed with ice-cold 1xDPBS. Cells were centrifuged and the supernatant was discarded. Cells were immediately resuspended in ice-cold lysis buffer (10 mM Tris–HCl, pH 7.4, 10 mM NaCl, 3 mM MgCl2 and 0.1% IGEPAL CA-630) and centrifuged at 500×*g* for 10 min. The supernatant was aspirated, and pellets were resuspended in transposase reaction mix (25 µL 2xTD Buffer, 2.5 µL transposase (Illumina), and 22.5 µL nuclease-free water). The transposition reaction was carried out at 37 ˚C for 30 min at 600 rpm in a shaking thermomixer (Eppendorf). Immediately after completion of the transposition reaction, the samples were purified using the MinElute Reaction Clean up kit (Qiagen) and eluted into 10 µL of EB. Samples were stored at – 20 ˚C until PCR amplification step. PCR amplification was performed as previously described [8] using custom Nextera primers. After initial amplification, a portion of the samples were run on qPCR (ViiA7 Applied Biosystems) to determine the additional number of cycles needed for each library. The libraries were purified using the MinElute Reaction Clean up kit (Qiagen), eluted into 20 µL EB and then size selected using AmpureXP(Beckman-Coulter) beads at a ratio of 1.8:1 beads/sample, and eluted into 40 µL of nuclease-free water. Library size distribution was determined by Bioanalyzer (Agilent) capillary electrophoresis and library concentration was determined by Qubit 3 (Life Technologies). Quality of libraries was checked by shallow sequencing (1 million raw reads) on a Miseq (Illumina) at 75 × 75 paired-end sequencing. Those libraries that appeared to have size distributions similar to previous reports (Additional file 1: Figure S1) were pooled together and deep sequenced on a HiSeq2500 (Illumina) at 100 × 100 reads at the Vincent J. Coates Genomics Sequencing Laboratory at UC Berkeley.

### Data processing

Demultiplexed sequencing data were processed using the ENCODE ATAC-seq pipeline version 1.1.6 and 1.4.2 (https://github.com/ENCODE-DCC/atac-seq-pipeline) using the mm10 assembly and the default parameters. In version 1.4.2 changed: atac.multimapping = 0, atac.smooth_win = 150, atac.enable_idr = true, atac.idr_thresh = 0.1 to be consistent with the mapping/peak calling performed with previous versions.

Peak filtering, hierarchical clustering, and tSNE plot production were performed using the chromVAR package (https://github.com/GreenleafLab/chromVAR). First, the optimal peak-list from the IDR output for each cell type was concatenated and sorted, then used as the peak input for chromVAR. The blacklist filtered bam files for reach replicate was used as input along with the sorted peak file. The fragment counts in each peak for each replicate and GC bias was calculated, and then the peaks were filtered using filterPeaks function with the default parameters and non-overlapping = TRUE. The master peak-list was extracted at this point, which contained 84,243 peaks, and used throughout the study. The deviations were calculated using every peak, and the tSNE and correlation functions were also performed using the deviations output and the default parameters.

Annotation of peaks, generation of histogram plot, merging of peaks, and motif enrichment were performed by HOMER (http://homer.ucsd.edu/homer/). Peaks were annotated using the annotatePeaks.pl function with the mm10 assembly and default parameters. Histogram was created by first shifting the bam files using DeepTools alignmentSieve.py with the flag –ATACshift. Next, tag directories were made using the Tn5 shifted bam files using HOMER makeTagDirectory. The histogram was made using the annotatePeaks.pl function with the default settings and the flags: -size -500,500 and -hist 5. Peak lists were compared using the mergePeaks.pl function with default settings and the flags -d given, -venn, and for the unique peak-lists -prefix. Motif enrichment was performed using the findMotifsGenome.pl package with default parameters using the flag -size given and custom background peaks, which consisted of the combination of all the peak-lists for the cell types not being analyzed. Instances of motifs in non-promoter peaks were found by using the annotatePeaks.pl function with the -m flag, using custom made motif files for each cell type containing the top 10 enriched motifs found.

The GREAT tool (http://great.stanford.edu/public/html/) was used to annotate non-promoter peaks to target genes. The peak-lists were reduced to BED4 files from the HOMER annotations output and used as input. The whole mm10 genome was used as the background regions, and the association rule settings were set as Basal plus extension, proximal window 2 kb upstream, 1 kb downstream, plus distal up to 1 Mb and included curated regulatory domains. All genome track visualizations were made using the UCSC genome browser. Graphs were made in either Microsoft Excel or GraphPad Prism 8. Annotations to figures were performed using Adobe Illustrator CC and Adobe Photoshop CC.

ChIP data were handled as follows: the enhancer list from [27] was mapped to mm10 using the liftOver tool, then compared to the master peak-list. The raw sequencing data for H3K4me1 and H3K27Ac in LT-HSCs were downloaded from GEO and mapping to mm10 and peak calling were performed using the parameters listed in the publication [27].

## Supplementary information

**Additional file 1: Figure S1.** Library fragment distributions for ATAC-seq samples. The library size distribution after deep-sequencing, mapping, and filtering to unique reads is shown of both replicates for **A**) HSCs **B**) EPs **C**) MkPs **D)** GMs **E**) B cells, and **F**) T cells.

**Additional file 2: Figure S2.** Erythroid-selective accessibility of the β-globin cluster. ATAC-seq signal tracks of the six cell types in this study at the β-globin cluster (chr7: 103,792,027–103,879,340; mm10). The adult globin genes β*-major (ßmaj)* and β*-minor (ßmin),* as well as the hypersensitive sites (HS1-4,6) of the Locus Control Region (LCR) that regulates expression of the genes in this locus, displayed accessibility in EPs. HS2, but not the other HSs, and the β-major promoter were also accessible in HSCs, possibly indicating a “permissive” chromatin state. Accessibility of HS2 and HS4 in MkPs may relate to a closer relationship to HSCs and/or EPs (Fig. 1). As expected, no accessibility was observed at the fetal-specific *epsilon Y globin (Ey), *β*-h1 (ßh1)*, β*-h2 (ßh2)* genes, or HS5. Likewise, GMs, B and T cells, that do not express β-globin genes, did not display accessibility of any of the regulatory elements in the locus.

**Additional file 3: Figure S3. **Lineage specific, HSC-primed peaks were marked by H3K4me1 and not H3K27Ac. **A**) About one in three (21,085 peaks out of 71,072) of our ATAC-seq non-promoter peaks in the master peak-list overlapped with peaks designated as probable enhancers based on H3K4me1 and H3K27Ac ChIP data (Lara-Astiaso et al.). **B**) About one in three (32.5%) of all HSC-primed peaks for the five unipotent lineage cell types were also marked by the histone modification H3K4me1, and 2.8% were marked by H3K27Ac. **C**) HSC-primed peaks for each unipotent lineage were primarily marked by H3K4me1 and not H3K27Ac. Results in panel B represent the aggregate of the results shown in panel C.

## Data Availability

The datasets generated during and/or analyzed during the current study are available in the Gene Expression Omnibus (GEO), accession number GSE162949.
