# Supplementary figures and images for: Chromatin accessibility maps provide evidence of multilineage gene priming in hematopoietic stem cells
Source: Epigenetics Chromatin. 2021 Jan 6;14:2. doi: 10.1186/s13072-020-00377-1 (PMC7789351; doi:10.1186/s13072-020-00377-1)

Supplemental Figure 1

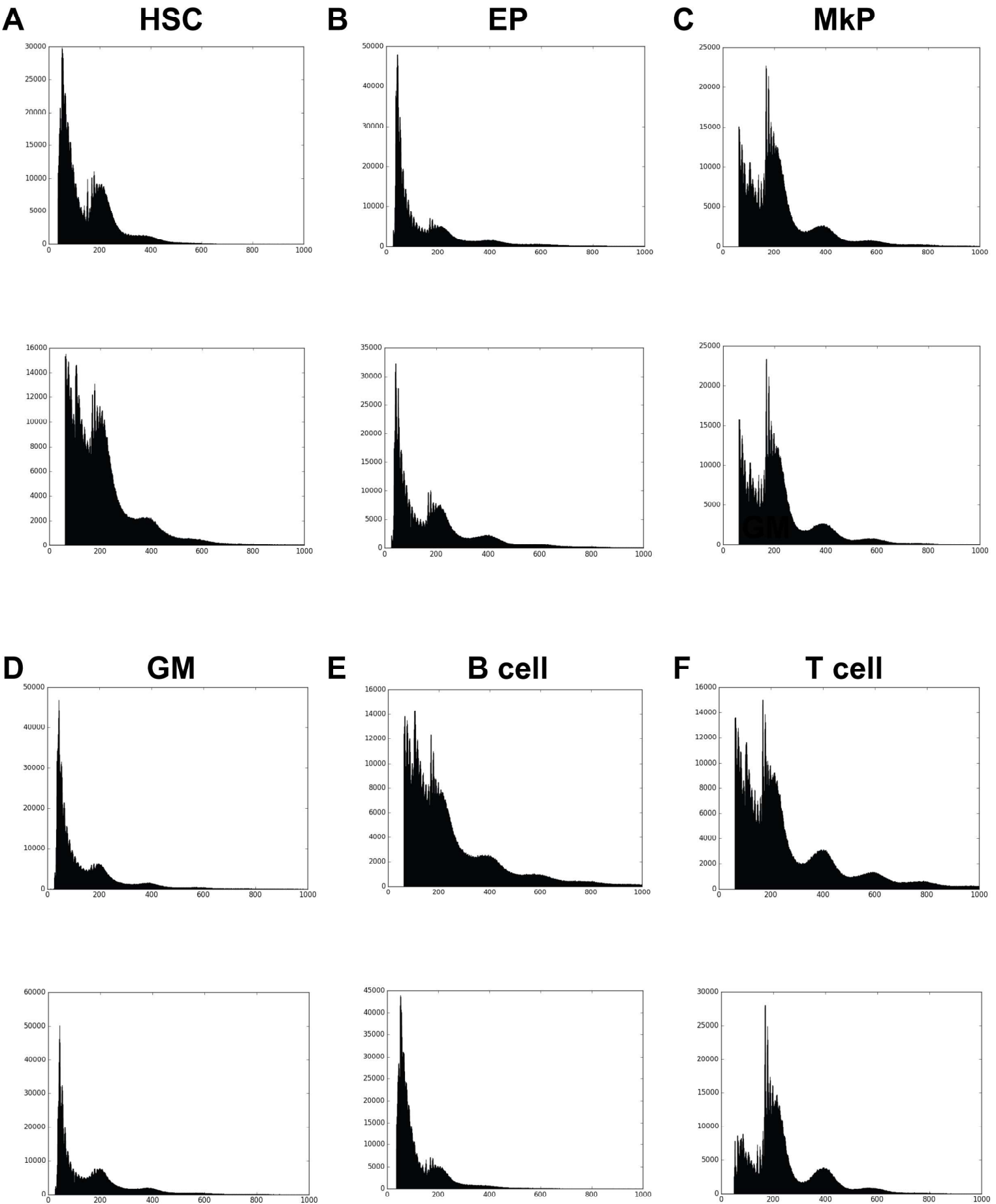

Supplement: Supplementary file 1 — Additional file 1: Figure S1. Library fragment distributions for ATAC-seq samples. The library size distribution after deep-sequencing, mapping, and filtering to unique reads is shown of both replicates for A) HSCs B) EPs C) MkPs D) GMs E) B cells, and F) T cells. [file 13072_2020_377_MOESM1_ESM.pdf]

# Supplemental Figure 2

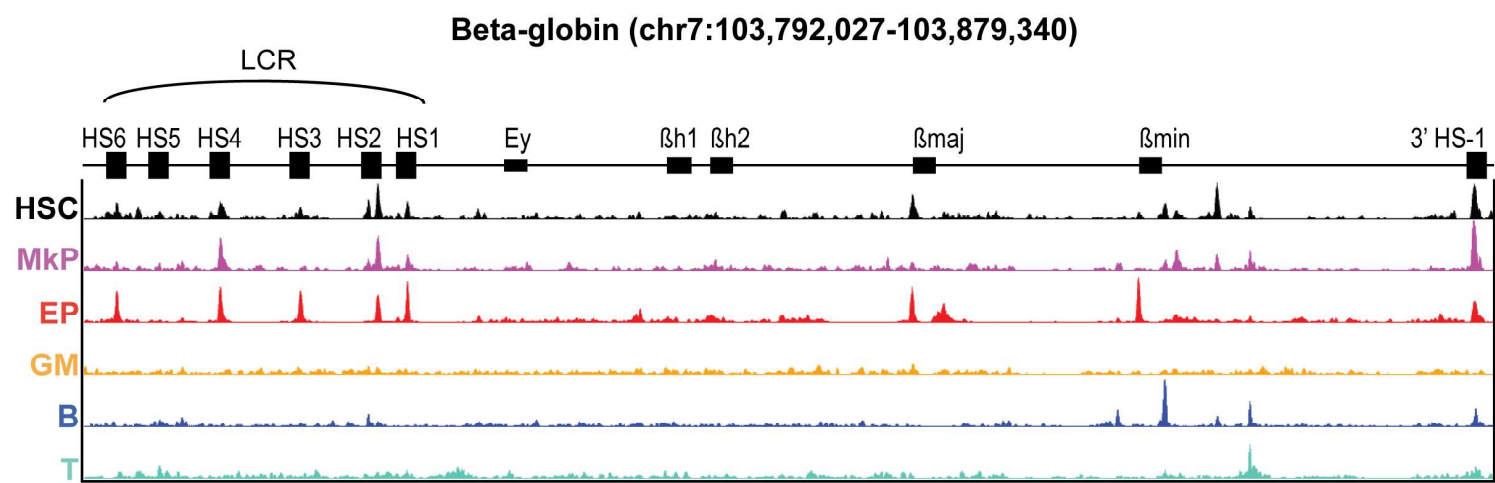

Supplement: Supplementary file 2 — Additional file 2: Figure S2. Erythroid-selective accessibility of the β-globin cluster. ATAC-seq signal tracks of the six cell types in this study at the β-globin cluster (chr7: 103,792,027–103,879,340; mm10). The adult globin genes β-major (ßmaj) and β-minor (ßmin), as well as the hypersensitive sites (HS1-4,6) of the Locus Control Region (LCR) that regulates expression of the genes in this locus, displayed accessibility in EPs. HS2, but not the other HSs, and the β-major promoter were also accessible in HSCs, possibly indicating a “permissive” chromatin state. Accessibility of HS2 and HS4 in MkPs may relate to a closer relationship to HSCs and/or EPs (Fig. 1). As expected, no accessibility was observed at the fetal-specific epsilon Y globin (Ey), β-h1 (ßh1), β-h2 (ßh2) genes, or HS5. Likewise, GMs, B and T cells, that do not express β-globin genes, did not display accessibility of any of the regulatory elements in the locus. [file 13072_2020_377_MOESM2_ESM.pdf]

# Supplemental Figure 3

A

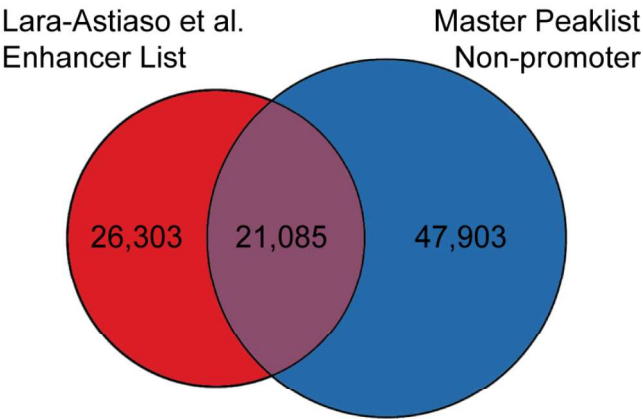

B

ATAC-seq Peak Overlap

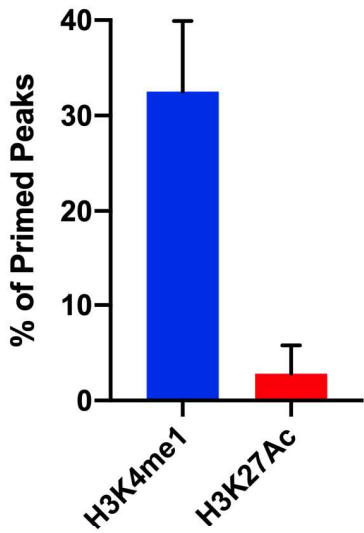

C

Peak Overlap with  
ChIP Peaks

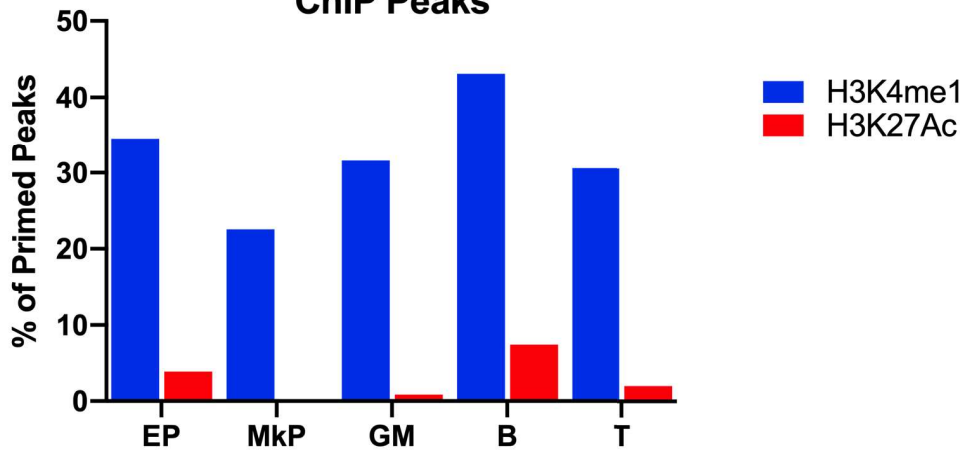

Supplement: Supplementary file 3 — Additional file 3: Figure S3. Lineage specific, HSC-primed peaks were marked by H3K4me1 and not H3K27Ac. A) About one in three (21,085 peaks out of 71,072) of our ATAC-seq non-promoter peaks in the master peak-list overlapped with peaks designated as probable enhancers based on H3K4me1 and H3K27Ac ChIP data (Lara-Astiaso et al.). B) About one in three (32.5%) of all HSC-primed peaks for the five unipotent lineage cell types were also marked by the histone modification H3K4me1, and 2.8% were marked by H3K27Ac. C) HSC-primed peaks for each unipotent lineage were primarily marked by H3K4me1 and not H3K27Ac. Results in panel B represent the aggregate of the results shown in panel C. [file 13072_2020_377_MOESM3_ESM.pdf]
